# 

**Published:** 1887-09

**Authors:** 


### BOOK NOTICES.

ROMANTIC LOVE AND PERSONAL BEAUTY, THEIR DEVELOPMENT, CASUAL RELATIONS, HISTORIC AND NATIONAL PECULIARITIES.—The above is the comprehensive title of a recent volume of five hundred and fifty pages by Henry T. Fink, and from an examination of its contents, we are forced to admit that the author has with great thoroughness, covered his ground. The several branches of his general subject are exhaustively treated, traditionally and historically, in respect to tribes and peoples, and even animals ; as well as sentimentally, poetically and physiognomically, in portraying the varieties of expression of which the sublime passion is capable, in its different moods.

In a physiological sense, the book has great value, especially as a guide to health, whilst many of the foibles which mar the social state, not only of dress, but manners, are pointed out with discriminating sarcasm. The volume shows that the author's leading motive was to impart a class of information which would furnish the groundwork of needed reforms, rather than to make a book, and his pages illustrate extensive reading and great painstaking in their preparation.

We cannot too highly recommend this book as a manual of physical beauty and good taste in dress. Published and sold by Macmillan & Co., 112 4th Ave, New York City. Price $2.

WE HAVE RECEIVED, with compliments of M. LeMarquand (Paris and New York), the Report of the Maryland State Board of Health, 1887 ; a quarto of 176 pp., containing much valuable information respecting the sanitation of Cities.

VICK'S ILLUSTRATED MONTHLY for August shows no falling off in either text or illustrations. To the amateur gardener it will be found invaluable. Published by James Vick, Seedsman, Rochester, N. Y., at $1.25 a year.

HORTICULTURAL ART JOURNAL for August, beautifully illustrated, is upon our table. Published by Stecher Lithographic Co., Rochester, N. Y., at $3.00 a year.

Our thanks are due to the following named surgeons and physicians for valuable treatises:

J. McC. Perkins, Esq., Boston and Washington, D. C.

Pamphlet on the subject of "Letters Patent," of value to inventors.

J. H. Kellogg, M. D., Battle Creek, Mich.

"Radical Cure of Retro-Displacements," etc.

C. W. Moore, M. D., San Francisco.

"Advances in Surgery, Medicine," etc., "in the last forty years."

G. W. H. Kemper, M. D., Muncie, Ind.

"Practical thoughts for Physicians."

E. F. Ingals, A. M. M. D., Chicago.

"Suppurative Inflammation," etc., and "Intubation of the Larynx."

J. K. Brown, Commissioner.

Third Annual Report of the New York State Dairy Commission. Two hundred and thirty pages, containing much valuable information upon milk and its products of butter and cheese, and their pernicious adulteration.

We have received the August number of the *Boston Journal of Health*, a new monthly publication which began life with its July issue. If we were to judge of its usefulness as a guide to health, from the number before us, we should say that it promises to be of great value to its patrons, and we are glad to welcome it among our exchanges. Published at Boston, at one dollar a year, and for sale by the leading News companies, Boston and New York.

### AN ERROR POINTED OUT AND CORRECTED.

Ithaca, N. Y., August 3, 1887.

Editor, Hall's Journal of Health.

Sir:—If Mr. John Franklin Clark will try the experiment he describes on page 183, current vol. of the Journal, he will upset his theory. Such behavior as he claims for the bar, A is possible only when its bearing is *above* its centre of gravity.

J. H. Sherman.

Editor of, Hall's Journal of Health.

Sir:—In your August issue an article by me entitled, "Equilibrium the controlling Force in Nature" was published, for which I owe an apology to you, and to the readers of the Journal.

While stoping at a hotel in Boston last summer, the experiment was tried with a long, flat and thin bar, found in the room, and the article as published was written, the figures being drawn to illustrate it.

One of your correspondents having pointed out to you, that the effect stated in the article can *only occur* when *the bearing upon the fulcrum is above the center of gravity of the bar*, I desire to state, that I have by experiment with a square straight bar of iron, demonstrated the correctness of your correspondents statement, and thus publicly acknowledge the error into which I was led by not observing a deflection in the bar used in my first experiment.

John Franklin Clark.

